# Wideband Spectrum Sensing Based on Single-Channel Sub-Nyquist Sampling for Cognitive Radio

**DOI:** 10.3390/s18072222

**Published:** 2018-07-10

**Authors:** Changjian Liu, Houjun Wang, Jie Zhang, Zongmiao He

**Affiliations:** 1 School of Automation Engineering, University of Electronic Science and Technology of China, Chengdu 611731, China; hjwang@uestc.edu.cn (H.W.); zhangjie303006@126.com (J.Z.); 2 National Key Laboratory of Science and Technology on Communications, University of Electronic Science and Technology of China, Chengdu 611731, China; hezongmiao@std.uestc.edu.cn

**Keywords:** cognitive radio, spectrum sensing, single-channel sub-Nyquist sampling, multi-coset sampling, sampling pattern design, minimal circular sparse ruler

## Abstract

Spectrum sensing is an important task in cognitive radio. However, currently available Analog-to-Digital Converters (ADC) can hardly satisfy the sampling rate requirement for wideband signals. Even with such an ADC, the cost is extremely high in terms of price and power consumption. In this paper, we propose a spectrum-sensing method based on single-channel sub-Nyquist sampling. Firstly, a serial Multi-Coset Sampling (MCS) structure is designed to avoid mismatches among sub-ADCs in the traditional parallel MCS. Clocks of the sample/hold and ADC are provided by two non-uniform sampling clocks. The cooperation between these two non-uniform sampling clocks shifts the high sampling rate burden from the ADC to the sample/hold. Secondly, a power spectrum estimation method using sub-Nyquist samples is introduced, and an efficient spectrum-sensing algorithm is proposed. By exploiting the frequency-smoothing property, the proposed efficient spectrum-sensing algorithm only needs to estimate power spectrum at partial frequency bins to conduct spectrum sensing, which will save a large amount of computational cost. Finally, the sampling pattern design of the proposed serial MCS is given, and it is proved to be a minimal circular sparse ruler with an additional constraint. Simulations show that mismatches in traditional parallel MCS have a serious impact on spectrum-sensing performance, while the proposed serial MCS combined with the efficient spectrum-sensing algorithm exhibits outstanding spectrum-sensing performance at much lower computational cost.

## 1. Introduction

With the increase of wireless communication, spectrum resources have become increasingly scarce. However, most primary users, or authorized users, do not use the spectrum resources allocated to them most of the time [1]. For example, RFeye node [2] is used to monitor the wireless spectrum at Queen Mary University of London, and most of the spectrum resources are found to be idle [3]. TeleVision White Space (TVWS) in the Ultra High Frequency (UHF) band is the spectrum band not used by Digital Terrestrial Televisions (DTT), and Programme Making and Special Events (PMSE). The wireless communication network using TVWS can cover above 10 km in diameter, and it has strong penetration capabilities for buildings, mountains and forests. Cognitive radio devices working in TVWS can be used in rural areas where the communication infrastructure is imperfect to build a communication link among machines and improve residents’ access to the Internet. The communication regulator in the United Kingdom, the Office of Communications (Ofcom), announced that using TVWS does not need a communication license in December 2015 [4]. Therefore, TVWS is one of the most promising opportunistic spectrum access resources.

TVWS can be used by secondary users to transmit their information to enhance spectrum utilization efficiency. Geo-location database is a way to discover available TVWS [5]. However, this method requires a wired or wireless connection with the Geo-location database in advance [6]. The dynamic changes in the wireless environment pose a huge challenge to this database-based approach. Therefore, we need to quickly and accurately detect whether primary users exist before using spectrum resources in TV band. According to the well-known Nyquist sampling theorem, alias free sampling requires the system sampling rate be higher than twice the maximum frequency of the signal. The currently available Analog-to-Digital Converters (ADC) often cannot satisfy the sampling rate requirement for wideband signals. Even with such an ADC, the cost is usually extremely high in terms of price and power consumption. Thus, it is attractive to conduct spectrum sensing based on sub-Nyquist sampling techniques.

Some parallel sub-Nyquist sampling schemes have been developed in recent years, such as the Modulated Wideband Converter (MWC) [7] and the parallel Multi-Coset Sampling (MCS) [8]. A Distributed Modulated Wideband Converter (DMWC) scheme is proposed, and a sensor node is considered to be an MWC channel in DMWC [9]. DMWC combines cooperative spectrum sensing and MWC techniques for wideband spectrum sensing. A Random Triggering-based Modulated Wideband Compressive Sampling (RT-MWCS) is also designed for repetitive sparse wideband signals [10]. However, the input signal is multiplied with the periodic Pseudo-Random Binary Sequence (PRBS) by a mixer in MWC, and it is difficult to suppress the unwanted harmonics due to the usage of PRBS instead of a single-tone as the local oscillator signal. Parallel MCS is another promising sub-Nyquist sampling scheme which has several sub-ADCs working parallelly. The sampling clocks of these sub-ADCs have the same frequency but different phases. Time-Interleaved ADC (TIADC) platform can be used to implement parallel MCS to further improve the time resolution of TIADC. Just as mismatches among sub-ADCs, mainly offset mismatch, gain mismatch and sampling clock skew, degrade the Spurious Free Dynamic Range (SFDR) of a TIADC system [11,12], they can also severely deteriorate spectrum-sensing performance based on parallel MCS.

The mismatch problem in parallel MCS can be avoided in single-channel sampling structure. A single-channel Non-Uniform Sampling (NUS) structure is design, in which a custom-designed Sample/Hold (S/H) and a commercial ADC are used [13]. Then a nonlinear compressive sensing algorithm is used to reconstruct the signal. The computational complexity of reconstruction is reduced by emulating MCS in NUS [14]. The signal sparsity is exploited to reduce the sampling rate requirement in [7,8,13,14], and the Nyquist sampling rate is required when the signal is not sparse.

The above mentioned sub-Nyquist sampling methods concentrate on signal reconstruction, while one may only be interested in the power spectrum in some practical applications, such as cognitive radio and spectrum monitoring. A Single-Channel Modulated Wideband Converter (SCMWC) scheme is proposed, and experiment circuit is designed using commercial Integrated Circuits (ICs) to verify the feasibility of SCMWC [15]. Only power spectrum information is estimated to conduct spectrum sensing in [15]. The signal statistical property is exploited to estimate finite resolution power spectrum of signal without requiring the signal to be sparse [16]. The mean and variance of the finite resolution power spectrum estimator are investigated when the number of sub-Nyquist samples is limited [17]. The signal statistical property is also utilized to estimate power spectrum of signal in [18]. After the power spectrum is obtained, the primary user is detected using the energy detection method. However, the estimation of power spectrum requires a large amount of calculation. Compared with the multi-band model [18], authors in [19] consider both multi-band and multi-tone models, the case of sparse and non-sparse, and known and unknown carrier frequencies. They derive minimum sampling rate requirement for each setting without noise and provide power spectrum reconstruction techniques to achieve these minimum sampling rates. The least squares method is used to estimate the signal power spectrum, and the full rank condition of the system matrix is viewed as a circular sparse ruler design problem [20,21].

In this paper, we propose a spectrum-sensing method based on single-channel sub-Nyquist sampling. To begin with, to avoid the mismatch problem among sub-ADCs in parallel MCS [16,17,18], a serial MCS structure is proposed. The proposed serial MCS mainly consists of a S/H, an ADC and two different periodic non-uniform sampling clocks. The clocks of S/H and ADC are provided by these two different periodic non-uniform sampling clocks. The S/H functions similar to an analog signal register, which can shift the high sampling rate requirement for wideband signals from ADC to S/H. Although the sampling rate requirement for the ADC in serial MCS is higher than that in parallel MCS, there is no mismatch problem and the implementation size is smaller. Moreover, to reduce the computational complexity in [18], we propose an efficient spectrum-sensing algorithm. The frequency-smoothing property of the power spectrum estimator is exploited to estimate the frequency-smoothed power spectrum of the signal. Since the frequency bins in a frequency-smoothing window are highly correlated, spectrum sensing only needs estimate power spectrum at partial frequency bins, which will save a large amount of computational cost. Then finally, to use the least square algorithm to estimate the power spectrum, we must ensure that the system matrix has full column rank. Whether the system matrix has full column rank is determined by the sampling pattern design, which is further proved to be a minimal circular sparse ruler design problem with an additional constraint. The sampling pattern design method in [20,21] is only suitable for the traditional parallel MCS. We consider the sampling pattern design for the proposed serial MCS. Simulations show that mismatches among sub-ADCs deteriorate the spectrum-sensing performance seriously, especially the false alarm probability, while the proposed MCS combined with the proposed efficient spectrum-sensing algorithm not only has outstanding spectrum-sensing performance, but also requires less computational load.

This paper is organized as follows: Section 2 presents the signal model and forms the problem. The sampling structure and sampling process of the proposed serial MCS are described in Section 3. Section 4 firstly introduces a power spectrum estimation method and then proposes an efficient spectrum-sensing algorithm. The sampling pattern design of the proposed serial MCS is developed in Section 5. Simulation results are given in Section 6. Simulations take some practical parameters into account, such as Signal-to-Noise Ratio (SNR), compression ratio, and acquisition time. Section 7 discusses using the spectrum-sensing module to receive communication signals.

## 2. Signal Model and Problem Formulation

The spectrum resource we are interested in is the UHF TV band, and its frequency range is from 470 MHz to 790 MHz. The bandwidth of a channel is 8 MHz, and there are 40 channels in total. Assume that M users are active during spectrum sensing. M users may comprise of both primary users and other secondary users. Without loss of generality, the *k*-th user can be represented as:

(1) $$u_{k}\left(t\right)=\sum_{n\in \mathbb{Z}}d_{k}\left[n\right]g_{k}\left(t-nT_{k}\right)e^{j2\pi f_{k}t}\;,\;k=1,2,\ldots ,M,$$

where $\left\{d_{k}\left[n\right]\right\}$ is a sequence of transmitted symbol, $g_{k}\left(t\right)$ is the pulse-shaping function, $T_{k}$ is the symbol duration and $f_{k}$ is the carrier frequency.

The practical wireless transmission channel mainly has two effects on the signal, one is log-normal shadowing which is a typical large-scale fading, the other is Rayleigh fading which is a typical small-scale fading [22,23,24]. To begin with, Large-scale fading denotes the average signal power attenuation caused by a wide range of movements. This kind of fading is mainly caused by the prominent terrain between the transmitter and the receiver. Experimental results show that the large-scale fading can be represented by a log-normal random variable [23]. Thus, large-scale fading can be expressed as:

(2) $$PL=PL_{0}+10\gamma \log\frac{d}{d_{0}}+X_{\rho }$$

where the reference distance $d_{0}$ is in the far field of the transmitting antenna, the reference power $PL_{0}$ is usually obtained by field test, the path loss exponent γ depends on the carrier frequency, antenna height and transmission environment, $X_{\rho }$ denotes a Gaussian random variable with variance $\rho^{2}$ and unit dB. Thus, the received signal affected by large-scale fading can be represented as:

(3) $$s_{k}\left(t\right)=10^{-PL_{k}/20}\cdot u_{k}\left(t\right)$$

where $PL_{k}$ denotes the attenuation between the k-th primary user and the cognitive radio device. Moreover, the primary user may arrive at the cognitive radio device via multiple paths, which can be modeled as Rayleigh fading. In other words, the envelope of the signal subjects to the Rayleigh distribution. The probability density function of Rayleigh distribution can be expressed as:

(4) $$p_{h}\left(r\right)=\{\begin{array}{l} \frac{r}{\lambda^{2}}e^{-r^{2}/2\lambda^{2}},\;r\geq 0\\ 0,\;\mathrm{otherwise} \end{array}$$

where r denotes the amplitude of the envelop of the received signal, $2\lambda^{2}$ denotes its average power. The k-th primary user $s_{k}\left(t\right)$ arrived at the cognitive radio device can be written as

(5) $$s_{k}\left(t\right)=10^{-PL_{k}/20}\cdot u_{k}\left(t\right)*h_{k}\left(t\right),$$

where ∗ and $h_{k}\left(t\right)$ denote the convolution operator and channel impulse response.

**Figure 1:**
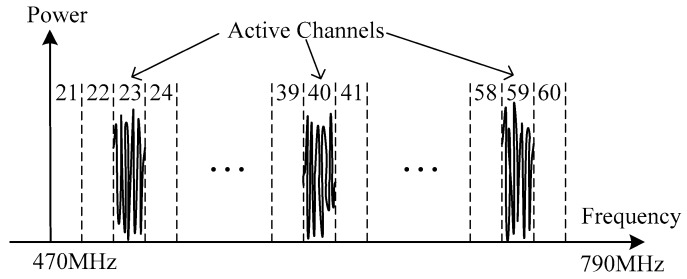
TV band with three users.

Then the signal received by the cognitive radio device is:

(6) $$x\left(t\right)=s\left(t\right)+n\left(t\right)=\sum_{k=1}^{M}s_{k}\left(t\right)+n\left(t\right),$$

where s(t) is the signal component and n(t) is the additive white Gaussian noise. Figure 1 shows a wideband signal with 3 active users. They occupy the 23rd, 40th and 59th channel, while the other channels are vacant. These vacant channels can be used by secondary users to transmit their information.

After the spectrum range and channel partitioning format in the UHF TV band is given, the problem of wideband spectrum sensing is to detect which channels are occupied by the primary users and which channels are vacant. A band-pass filter is firstly used to filter out the noise outside the band [470 MHz, 790 MHz], and then a mixer is utilized to demodulate the filtered signal. The demodulated signal is a band-limited signal and its bandwidth is 320 MHz. A traditional method to solve this problem is to first sample the demodulated signal at the Nyquist sampling rate, and then estimate its power spectrum. Finally, the energy detection method is used to determine whether primary users exist. The Nyquist sampling theorem states that alias-free sampling requires the sampling rate be higher than twice the maximum frequency of the signal. Therefore, the sampling rate requirement for the demodulated signal is 640 MHz, which poses a great challenge to current available ADCs. Even with such a high sampling rate ADC, it is usually unacceptable in terms of price and power consumption for some sensors and battery-powered devices. In this paper, we consider using sub-Nyquist sampling technique to conduct spectrum sensing.

## 3. Single-Channel Sub-Nyquist Sampling Structure

**Figure 2:**
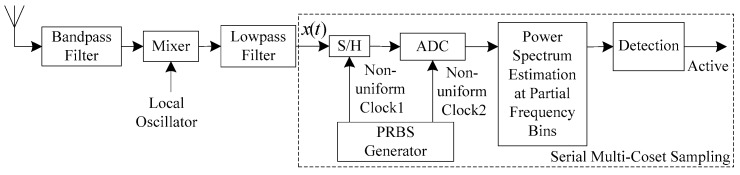
The block diagram of single-channel sub-Nyquist sampling.

An overview of the proposed single-channel sub-Nyquist sampling scheme is shown in Figure 2. The signal received by the antenna is first band-pass filtered to remove the out-of-band noise and interference, and then a mixer is used to demodulate the filtered signal. Finally, low-pass filter is used to remove the local oscillator signal. The demodulated signal still has a high bandwidth if we are interested in many channels. The dotted box shows the proposed serial MCS. The S/H is used to sample and temporarily store the input analog signal x(t). Then the subsequent ADC can sample it at a lower rate. Two periodic Pseudo-Random Binary Sequences are used as the non-uniform sampling clocks of S/H and ADC. The periodic non-uniform sampling clock provided to S/H is determined by the sampling pattern of MCS, while the periodic non-uniform sampling clock provided to ADC is dependent on not only the sampling pattern but also the sampling rate of ADC. The spectrum-sensing procedure can be summarized as follows: (1) Classifying the periodic non-uniformly sampled data into several uniform sampling sequences; (2) Power spectrum at partial frequency bins is estimated using these uniform sampling sequences; (3) Compare the estimated power spectrum with a pre-defined threshold to determine whether the primary user exists.

In detail, let x(t) denote the signal output from the low-pass filter. Let the Nyquist sampling rate and interval of x(t) be $f_{N}$ and T, respectively. Let the sampling rate and interval of ADC be $f_{\mathrm{ADC}}$ and $T_{\mathrm{ADC}}$, respectively. Let d represent the sub-Nyquist sampling factor which is defined as $d=f_{N}/f_{\mathrm{ADC}}$. q samples are selected from L consecutive Nyquist samples in MCS. The sampling pattern $C=\left\{c_{1},c_{2},\ldots ,c_{q}\right\}$, where $1\leq c_{1}<c_{2}<\ldots <c_{q}\leq L$ is used as the sample index. Let $N_{0}$ represent the number of samples of a coset, and the total number of periodic non-uniformly sampled data is $N=q\times N_{0}$. We will explain in Section 5, Sampling pattern design, that at least one pair of cosets with only 1×T time difference should be included in the sampling pattern. The ADC cannot directly capture two samples $c_{i}$ and $c_{i+1}$ with only 1×T time difference because the sampling interval of ADC $T_{\mathrm{ADC}}=d\times T$ is greater than 1×T. The function of S/H is to temporarily store the second coset $c_{i+1}$ until the ADC has finished capturing the previous coset $c_{i}$.

**Figure 3:**
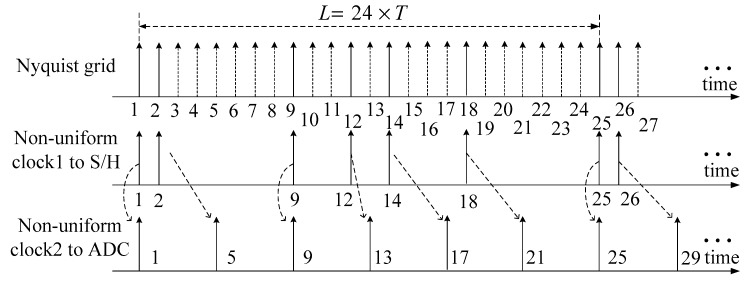
The sampling process of serial MCS.

An example of the sampling process is given in Figure 3, where q=6, L=24, $f_{\mathrm{ADC}}=f_{N}/4$ and C={1,2,9,12,14,18}. The first coset C(1) can be sampled directly, while the second coset C(2) must be firstly sampled by the S/H and subsequently captured by the following ADC on the fifth Nyquist time grid. Thus, the third coset C(3) must be greater than or equal to 5, otherwise, it will lose the last coset C(2). The cooperation between the first and the second periodic non-uniform sampling clock ensures that two adjacent cosets with only 1×T time difference can be captured by an ADC with sampling rate $f_{\mathrm{ADC}}$ which is much lower than $f_{N}$, and this is the essence of the proposed serial MCS.

The periodic non-uniformly sampled data are classified into several uniform sampling sequences. Let y[n],1≤n≤N represent the periodic non-uniformly sampled data output from the ADC and it can be written as:

(7) $$y\left[n\right]=\left[y_{c1}\left[1\right],y_{c2}\left[1\right],\ldots ,y_{cq}\left[1\right],y_{c1}\left[2\right],\ldots ,y_{cq}\left[N_{0}\right]\right],$$

where $y_{ci}\left[k\right],\;1\leq i\leq q,\;1\leq k\leq N_{0}$ is the sample obtained from the *k*-th Nyquist sample block with length *L* using sample index C(i). Please note that the number of y[n] equals to the total number of periodic non-uniformly sampled data N. Then periodic non-uniformly sampled data y[n],1≤n≤N is classified into q uniform sampling sequences

(8) $$y_{ci}\left[k\right]=y\left[\left(k-1\right)\times q+i\right],\;1\leq k\leq N_{0},\;1\leq \;i\leq q.$$

Let $y_{ci}={\left[y_{ci}\left[1\right],y_{ci}\left[2\right],\ldots ,y_{ci}\left[N_{0}\right]\right]}^{T}$ represent the *i*-th uniform sampling sequence which corresponds to the *i*-th coset $c_{i}$. The sampling rate and time offset of $y_{ci}$ are $f_{N}/L$ and ci×T, respectively. q uniform sampling sequences can be written in matrix form concisely:

(9) $$y_{c}={\left[y_{c1},y_{c2},\ldots ,y_{cq}\right]}^{T}.\;$$

The parameter of current available ICs should be considered in practical implementation. The sampling rate requirement for S/H is high, but the sampling rate requirement for the following ADC is low. The high sampling rate burden for wideband signals is shifted from ADC to S/H. The sampling rate and input bandwidth of current state-of-the-art S/H are 4GSPS (Giga-Samples Per Second) and 18 GHz, respectively (HMC760LC4B, Analog Devices, Inc., Norwood, MA, USA). Moreover, the jitter of the periodic non-uniform sampling clock is also an important parameter in serial MCS. The alternating rate and jitter of current state-of-the-art PRBS generator are 80 GHz and 600 fs (femtosecond), respectively [25]. Periodic non-uniform sampling clock can also be generated using commercial parallel-to-serial converter. The alternating rate and typical jitter of current state-of-art parallel-to-serial converter are 3.2 GHz and 200 fs, respectively (MC100EP446, ON Semiconductor, Inc., Phoenix, AZ, USA).

## 4. Spectrum Sensing

After q uniform sampling sequences are obtained using the proposed serial MCS, they are further processed to conduct spectrum sensing. We firstly introduce a power spectrum estimation method [18], and then an efficient spectrum-sensing algorithm is proposed.

### 4.1. Power Spectrum Estimation

This section mainly consists of two parts:

(a) Establish the theoretical model for power spectrum estimation.
(b) Develop an implementation method for the theoretical model.

*(a). Establish the Theoretical Model for Power Spectrum Estimation*

Let X(f) denote the Fourier Transform of the demodulated signal x(t) in Figure 2. We first analyze the relationship between the spectrum of the *i*-th uniform sampling sequence $y_{ci}$ and the original spectrum X(f). Define $Y_{ci}\left(e^{j2\pi fLT}\right)$ as the Discrete Time Fourier Transform (DTFT) of the *i*-th uniform sampling sequence $y_{ci}$, and it can be written as:

(10) $$\begin{array}{lll} Y_{ci}\left(e^{j2\pi fLT}\right) & = & \sum_{n=-\infty }^{+\infty }y_{ci}\left[n\right]e^{-j2\pi fnLT}\\ & = & \frac{1}{LT}\sum_{l=-\frac{L}{2}}^{\frac{L}{2}-1}X_{ci}\left(f-\frac{l}{LT}\right)\\ & = & \frac{1}{LT}\sum_{l=-\frac{L}{2}}^{\frac{L}{2}-1}X\left(f-\frac{l}{LT}\right)e^{j2\pi c_{i}T\left(f-\frac{l}{LT}\right)}\\ & = & \frac{1}{LT}\sum_{l=-\frac{L}{2}}^{\frac{L}{2}-1}X\left(f-\frac{l}{LT}\right)e^{-j2\pi c_{i}\frac{l}{L}}e^{j2\pi fc_{i}T},\;f\in F_{0}, \end{array}$$

where $F_{0}=\left[0,1/\left(LT\right)\right)$ and L is assumed to be even. The band-limited property X(f)=0, |f|>1/(2T) is used in the derivation from the first step to the second step. The relationship between the spectrum of the delayed signal $X_{ci}\left(f\right)$ and the original spectrum X(f), $X_{ci}\left(f\right)=X\left(f\right)e^{j2\pi fc_{i}T}$, is used in the derivation from the second step to the third step. The term $e^{j2\pi fc_{i}T}$ in Equation (10) can be moved to the left of the equation, and define

(11) $$Y_{i}\left(e^{j2\pi fLT}\right)≜Y_{ci}\left(e^{j2\pi fLT}\right)e^{-j2\pi fc_{i}T}.$$

Equation (10) can be rewritten as

(12) $$Y_{i}\left(e^{j2\pi fLT}\right)=\frac{1}{LT}\sum_{l=-\frac{L}{2}}^{\frac{L}{2}-1}X\left(f-\frac{l}{LT}\right)e^{-j2\pi c_{i}\frac{l}{L}},\;f\in F_{0}.$$

At this point, we have established the relationship between the spectrum of the *i*-th uniform sampling sequence $y_{ci}$ and the original spectrum X(f) in Equation (12) for single uniform sampling sequence $y_{ci}$. q uniform sampling sequences, or the entire MCS system, can be expressed in matrix form concisely

(13) $$Y\left(e^{j2\pi fLT}\right)=Ax\left(f\right),\;f\in F_{0},$$

where $Y{\left(e^{j2\pi fLT}\right)}_{i}=Y_{i}\left(e^{j2\pi fLT}\right)$, $A_{il}=e^{-j2\pi c_{i}\frac{l}{L}}$, $x{\left(f\right)}_{l}=X\left(f-\frac{l}{LT}\right)$, 1≤i≤q and $-\frac{L}{2}\leq l\leq \frac{L}{2}-1$.

The demodulated signal x(t) comprises of signal component s(t) and noise component n(t). Let S(f) and N(f) denote the Fourier Transform of s(t) and n(t), respectively. Define L×1 vector $s{\left(f\right)}_{l}=S\left(f-\frac{l}{LT}\right),\;-\frac{L}{2}\leq l\leq \frac{L}{2}-1$ and L×1 vector $n{\left(f\right)}_{l}=N\left(f-\frac{l}{LT}\right),\;-\frac{L}{2}\leq l\leq \frac{L}{2}-1$. Let $R_{Y}\left(e^{j2\pi fLT}\right)$ denote the covariance matrix of $Y\left(e^{j2\pi fLT}\right)$, and it can be written as:

(14) $$R_{Y}\left(e^{j2\pi fLT}\right)=E\left\{Y\left(e^{j2\pi fLT}\right)Y^{H}\left(e^{j2\pi fLT}\right)\right\}.$$

Similarly, the covariance matrix of x(f), s(f) and n(f) can be expressed as $R_{x}\left(f\right)=E\left\{x\left(f\right)x^{H}\left(f\right)\right\}$, $R_{s}\left(f\right)=E\left\{s\left(f\right)s^{H}\left(f\right)\right\}$ and $R_{n}\left(f\right)=E\left\{n\left(f\right)n^{H}\left(f\right)\right\}$. Equation (14) can be further written as:

(15) $$R_{Y}\left(e^{j2\pi fLT}\right)=AR_{x}\left(f\right)A^{H}=A\left[R_{s}\left(f\right)+R_{n}\left(f\right)\right]A^{H}.$$

The following explanation shows that the covariance matrix $R_{x}\left(f\right)$ is a diagonal matrix. The (*i*,*j*)-th element of $R_{x}\left(f\right)$ can be expressed as:

(16) $$\begin{array}{lll} {\left[R_{x}\left(f\right)\right]}_{i,j} & = & E\left[x{\left(f\right)}_{i}x{\left(f\right)}_{j}^{*}\right]\\ & = & E\left\{\left[s{\left(f\right)}_{i}+n{\left(f\right)}_{i}\right]{\left[s{\left(f\right)}_{j}+n{\left(f\right)}_{j}\right]}^{*}\right\}\\ & = & E\left[s{\left(f\right)}_{i}s{\left(f\right)}_{j}^{*}\right]+E\left[n{\left(f\right)}_{i}n{\left(f\right)}_{j}^{*}\right]\\ & = & E\left[{\left|s{\left(f\right)}_{i}\right|}^{2}\right]\delta \left(i-j\right)+\sigma^{2}\delta \left(i-j\right), \end{array}$$

where the signal component s(t) and noise component n(t) are assumed to be independent, different primary users are assumed to be independent, and the property of stationary process is used in these derivations. The first term $E\left[{\left|s{\left(f\right)}_{i}\right|}^{2}\right]\delta \left(i-j\right)$ in Equation (16) corresponds to a diagonal matrix with only M non-zero elements, while the second term $\sigma^{2}\delta \left(i-j\right)$ in Equation (16) also corresponds to a diagonal matrix but with L non-zero elements. Therefore, the covariance matrix $R_{x}\left(f\right)$ is a diagonal matrix with only L non-zero diagonal elements. This characteristic is used to further develop the power spectrum estimation method.

Let Vec(·) and ⊗ represent the matrix vectorization operator and Kronecker product, respectively. Define $r_{Y}\left(e^{j2\pi fLT}\right)≜\mathrm{Vec}\left[R_{Y}\left(e^{j2\pi fLT}\right)\right]$, Equation (15) can be rewritten as:

(17) $$\begin{array}{lll} r_{Y}\left(e^{j2\pi fLT}\right) & = & \mathrm{Vec}\left[R_{Y}\left(e^{j2\pi fLT}\right)\right]\\ & = & \left[{\left(A^{H}\right)}^{T}⊗A\right]\mathrm{Vec}\left[R_{x}\left(f\right)\right]\\ & = & \left(A^{*}⊗A\right)\mathrm{Vec}\left[R_{x}\left(f\right)\right], \end{array}$$

where the matrix identity $\mathrm{Vec}\left(AXB\right)=\left(B^{T}⊗A\right)\mathrm{Vec}\left(X\right)$ is used. The vector form of the diagonal matrix $R_{x}\left(f\right)$ can be further written as $\mathrm{Vec}\left[R_{x}\left(f\right)\right]={\left[P_{1}\left(f\right)e_{1}^{T},P_{2}\left(f\right)e_{2}^{T},\ldots ,P_{L}\left(f\right)e_{L}^{T}\right]}^{T}$, where $P_{i}\left(f\right)$ is the *i*-th diagonal element and $e_{i}$ is selected from the *i*-th row of a L×L identity matrix. Further define ${\tilde{r}}_{x}\left(f\right)={\left[P_{1}\left(f\right),P_{2}\left(f\right),\ldots ,P_{L}\left(f\right)\right]}^{T}$ and a $L^{2}\times L$ selection matrix B which has 1 at the *j*-th column and the [(j−1)L+j]-th row. The vector form of the diagonal matrix $R_{x}\left(f\right)$ can be written as $\mathrm{Vec}\left[R_{x}\left(f\right)\right]={B\tilde{r}}_{x}\left(f\right)$. Equation (17) can be further expressed as

(18) $$\begin{array}{lll} r_{Y}\left(e^{j2\pi fLT}\right) & = & \left(A^{*}⊗A\right)\mathrm{Vec}\left[R_{x}\left(f\right)\right]\\ & = & \left(A^{*}⊗A\right){B\tilde{r}}_{x}\left(f\right)\\ & = & \Phi {\tilde{r}}_{x}\left(f\right), \end{array}$$

where $\Phi =\left(A^{*}⊗A\right)B$ is a $q^{2}\times L$ matrix. If Φ has full column rank and Equation (18) is an over-determined system, then least square algorithm can be used to estimate the power spectrum

(19) $${\tilde{r}}_{x}\left(f\right)=\Phi^{†}r_{Y}\left(e^{j2\pi fLT}\right),$$

where $\Phi^{†}$ is the Moore-Penrose inverse of Φ.

*(b). Develop an Implementation Method for the Theoretical Model*

The theoretical model of power spectrum estimation is established in the previous section. However, the Fourier transform involved in the theoretical model is implemented through Fast Fourier Transform (FFT) in practice. In this section, the implementation method of the theoretical model in the digital domain is discussed. The main contents include: (1) Compensate the fractional delay of the uniform sampling sequences in MCS. (2) Estimate the cross-power spectrum of the uniform sampling sequences using the frequency-smoothed cyclic cross-periodogram. (3) Detect the presence of the primary user using the estimated power spectrum.

To begin with, let $Y_{ci}\left[k\right]=Y_{ci}\left(e^{j2\pi fLT}\right){|}_{f=\frac{k}{LTN_{0}}},\;1\leq k\leq N_{0}$ represent the FFT of the *i*-th uniform sampling sequence. Define a constant phasor corresponding to the *i*-th uniform sampling sequence $P_{i}\left[k\right]=e^{-j2\pi fc_{i}T}{|}_{f=\frac{k}{LTN_{0}}},\;1\leq k\leq N_{0}$. Equation (11) in the theoretical model compensates the fractional delay of the *i*-th uniform sampling sequence of MCS. In practice, the implementation of fractional delay compensation in the digital domain can be represented as:

(20) $$Y_{i}\left[k\right]=P_{i}\left[k\right]Y_{ci}\left[k\right],\;1\leq k\leq N_{0}$$

where $Y_{i}\left[k\right]=Y_{i}\left(e^{j2\pi fLT}\right){|}_{f=\frac{k}{LTN_{0}}}$. Define x[n]≜x(nT) and its FFT $X\left[k\right]≜\frac{1}{\sqrt{N_{0}L}}\sum_{n=1}^{N_{0}L}x\left[n\right]e^{-j2\pi nk/N_{0}L}$. Partition X[k] into $X_{i}\left[k\right]≜X\left[k+\left(i-1\right)N_{0}\right]$, $1\leq k\leq N_{0}$ and 1≤i≤L. Define $x\left[k\right]={\left[X_{1}\left[k\right],X_{2}\left[k\right],\ldots ,X_{L}\left[k\right]\right]}^{T}$ and $Y\left[k\right]={\left[Y_{1}\left[k\right],Y_{2}\left[k\right],\ldots ,Y_{q}\left[k\right]\right]}^{T}$. Equation (13) in the theoretical model can be written as $Y\left[k\right]=Ax\left[k\right],1\leq k\leq N_{0}$.

Next, to estimate the signal power spectrum using Equation (19), we should first obtain $r_{Y}\left(e^{j2\pi fLT}\right)=\mathrm{Vec}\left[R_{Y}\left(e^{j2\pi fLT}\right)\right],\;f\in F_{0}$. The element at the *i*-th row and *j*-th column of matrix $R_{Y}\left(e^{j2\pi fLT}\right)$ is ${\left[R_{Y}\left(e^{j2\pi fLT}\right)\right]}_{i,j}=E\left\{Y{\left(e^{j2\pi fLT}\right)}_{i}Y{\left(e^{j2\pi fLT}\right)}_{j}^{*}\right\},\;f\in F_{0},1\;\leq i,j\leq q$. That is, in order to estimate the power spectrum of signal using Equation (19), we should first calculate the cross-power spectrum of $Y{\left(e^{j2\pi fLT}\right)}_{i}=Y_{i}\left(e^{j2\pi fLT}\right)$ and $Y{\left(e^{j2\pi fLT}\right)}_{j}=Y_{j}\left(e^{j2\pi fLT}\right)$ at frequency bin f, $f\in F_{0}$. A cross-power spectrum estimator [26] is introduced to calculate the cross-power spectrum of $Y_{i}\left(e^{j2\pi fLT}\right)$ and $Y_{j}\left(e^{j2\pi fLT}\right)$. Define $S_{Z_{i}Z_{j}}\left(\alpha ;f\right)≜E\left\{Z_{i}\left(f\right)Z_{j}^{*}\left(f-\alpha \right)\right\}$ as the cyclic cross-spectrum of $Z_{i}\left(f\right)$ and $Z_{j}\left(f\right)$, where α is the cyclic frequency. The frequency-smoothed cyclic cross-periodogram is used as a consistent estimator of $S_{Z_{i}Z_{j}}\left(\alpha ;f\right)$, and it can be written as

(21) $${\hat{S}}_{Z_{i},Z_{j}}\left(\alpha ;f\right)≜\frac{1}{N_{0}}\sum_{n=1}^{N_{0}}w\left(e^{j2\pi \left(f-\frac{n}{N_{0}LT}\right)LT}\right)Z_{i}\left(e^{j2\pi \frac{n}{N_{0}}}\right)Z_{j}^{*}\left(e^{j2\pi \left(\frac{n}{N_{0}LT}-\alpha \right)LT}\right),$$

where $Z_{i}\left(e^{j2\pi \frac{n}{N_{0}}}\right)=Z_{i}\left(e^{j2\pi fLT}\right){|}_{f=\frac{n}{N_{0}LT}}$, $Z_{i}\left(e^{j2\pi fLT}\right)$ is the DTFT of $z_{i}\left[n\right]$ and $w\left(e^{j2\pi fLT}\right)$ is the spectral window function. In practical digital implementation, FFT is used to calculate the DTFT. The frequency-smoothed cyclic cross-periodogram can be correspondingly written as

(22) $${\hat{S}}_{Z_{i},Z_{j}}\left(\alpha =\frac{m}{N_{0}LT};f=\frac{k}{N_{0}LT}\right)=\frac{1}{N_{0}}\sum_{n=1}^{N_{0}}w\left[k-n\right]Z_{i}\left[n\right]Z_{j}\left[n-m\right],$$

where $w\left[n\right]≜w\left(e^{j2\pi fLT}\right){|}_{f=n/N_{0}LT}$ and $Z_{i}\left[n\right]≜Z_{i}\left(e^{j2\pi fLT}\right){|}_{f=n/N_{0}LT}$. Define ${\left[R_{Y}\left[k\right]\right]}_{i,j}$ as ${\left[R_{Y}\left(e^{j2\pi fLT}\right)\right]}_{i,j}{|}_{f=\frac{k}{LTN_{0}}}=E\left\{Y_{i}\left(e^{j2\pi fLT}\right)Y_{j}^{*}\left(e^{j2\pi fLT}\right)\right\}{|}_{f=\frac{k}{LTN_{0}}}$, and it can be estimated using

(23) $${\left[R_{Y}\left[k\right]\right]}_{i,j}={\hat{S}}_{Y_{i},Y_{j}}\left(\alpha =0;f=\frac{k}{N_{0}LT}\right)=\frac{1}{N_{0}}\sum_{n=1}^{N_{0}}w\left[k-n\right]Y_{i}\left[n\right]Y_{j}^{*}\left[n\right].$$

Thus, the frequency-smoothed cyclic cross-periodogram of $R_{Y}\left[k\right]$ can be written as:

(24) $$\begin{array}{lll} {\hat{R}}_{Y}\left[k\right] & = & \frac{1}{N_{0}}\sum_{n=1}^{N_{0}}w\left[k-n\right]Y\left[n\right]Y^{H}\left[n\right]\\ & = & A\frac{1}{N_{0}}\sum_{n=1}^{N_{0}}w\left[k-n\right]x\left[n\right]x^{H}\left[n\right]A^{H}\\ & = & A{\hat{R}}_{x}\left[k\right]A^{H}, \end{array}$$

where ${\hat{R}}_{x}\left[k\right]=\frac{1}{N_{0}}\sum_{n=1}^{N_{0}}w\left[k-n\right]x\left[n\right]x^{H}\left[n\right]$. Define the diagonal elements of ${\hat{R}}_{x}\left[k\right]$ as ${\hat{\tilde{r}}}_{x}\left[k\right]=\mathrm{diag}\left({\hat{R}}_{x}\left[k\right]\right)$. Similar to Equation (19), we can estimate the power spectrum of signal using least square algorithm

(25) $${\hat{\tilde{r}}}_{x}\left[k\right]=\Phi^{†}{\hat{r}}_{Y}\left[k\right],\;k=1,2,\ldots ,N_{0},$$

where ${\hat{r}}_{Y}\left[k\right]=\mathrm{Vec}\left({\hat{R}}_{Y}\left[k\right]\right)$.

Finally, the spectrum-sensing problem is subsequently regarded as a binary hypothesis testing problem,

(26) $$\begin{array}{ll} H_{0}: & {\hat{\tilde{r}}}_{x}\left[k\right]\sim N\left(\sigma^{2},\rho^{2}\right)\\ H_{1}: & {\hat{\tilde{r}}}_{x}\left[k\right]\sim N\left(\sigma^{2}+S\left[k\right],\rho^{2}\right). \end{array}$$

The noise level $\sigma^{2}$ and estimate variance $\rho^{2}$ are assumed to be known, which can be obtained from receiver calibration, but the power spectrum of signal S[k] is unknown. Next, constant false alarm probability $P_{\mathrm{FA}}$ is imposed across all frequency bins, and the detection threshold is determined as

(27) $$\gamma =\rho Q^{-1}\left(P_{\mathrm{FA}}\right)+\sigma^{2},$$

where $Q^{-1}\left(\cdot \right)$ is the tail distribution function of the standard normal distribution.

### 4.2. Efficient Spectrum-Sensing Algorithm

The traditional spectrum-sensing method [18] first estimates the power spectrum of signal, and then uses the estimated power spectrum for primary user detection. However, the computational load of power spectrum estimation is heavy. The power spectrum estimation requires repeatedly solving Equation (25) $N_{0}$ times. Therefore, the power spectrum estimation requires a large amount of computation when the number of samples of a coset $N_{0}$ is large. Interestingly, note that the frequency-smoothed cyclic cross-periodogram is used to estimate ${\hat{R}}_{Y}\left[k\right]$ in Equation (24), and ${\hat{R}}_{x}\left[k\right]$ is also a frequency-smoothed result of the original $R_{x}\left[k\right]$. If the width of the spectral window function w[n] is wide enough, the estimated power spectrum ${\hat{\tilde{r}}}_{x}\left[k\right]=\mathrm{diag}\left({\hat{R}}_{x}\left[k\right]\right)$ at frequency bin k will contain the power spectrum information from the surrounding frequency bins k+1,k−1,k+2,k−2,…. In other words, the estimated power spectrum ${\hat{\tilde{r}}}_{x}\left[k\right]$ will reflect the outline information of the original power spectrum. Since the power spectrum information is highly correlated at frequency bins in the spectral window, it is not necessary to estimate the power spectrum information at all frequency bins. Therefore, to reduce computational complexity, only power spectrum at partial frequency bins is estimated to conduct spectrum sensing. The proposed efficient spectrum-sensing algorithm exploits the frequency-smoothing property of the power spectrum estimator to reduce computational complexity, and it is summarized as Algorithm 1.

**Algorithm 1.** Efficient Spectrum-Sensing Algorithm based on Power Spectrum at Partial Frequency Bins**Initialization:** Classify the periodic non-uniformly sampled data y[n] to q uniform sampling sequences $y_{ci}\left[k\right],1\leq \;i\leq q,\;1\leq k\leq N_{0}$, and calculate their FFT $Y_{ci}\left[k\right],\;1\leq i\leq q,\;1\leq k\leq N_{0}$. **Step 1:** Implement fractional delay compensation in the frequency domain, $Y_{i}\left[k\right]=Y_{ci}\left[k\right]\cdot P_{i}\left[k\right],\;1\leq i\leq q,\;1\leq k\leq N_{0}$, $P_{i}\left[k\right]=e^{-j2\pi c_{i}k/\left(LN_{0}\right)}$. **Step 2:** The width of the spectral window w[n] is chose to be $W_{\min}/△f$. Compute the frequency-smoothed cross-periodogram of $Y_{i}\left[m\right]$ and $Y_{j}\left[m\right]$
${\left\{{\hat{R}}_{Y}\left[m\right]\right\}}_{i,j}=\frac{1}{N_{0}}\sum_{n=1}^{N_{0}}w\left[m-n\right]Y_{i}\left[n\right]Y_{j}^{*}\left[n\right]$ at partial frequency bins $m=lN_{s},1\leq \;l\leq N_{0}/N_{s}$. **Step 3:** Estimate the power spectrum at partial frequency bins ${\hat{\tilde{r}}}_{x}\left[m\right]=\Phi^{†}{\hat{r}}_{Y}\left[m\right],\;\;m=lN_{s},1\leq \;l\leq N_{0}/N_{s}$. **Decision:** Compare ${\hat{\tilde{r}}}_{x}\left[m\right],\;\;m=lN_{s},1\leq \;l\leq N_{0}/N_{s}$ with the predefined threshold γ, and a sequence of binary results can be obtained. Then the result can be obtained by flipping consecutive 1s with length less than $W_{\min}/△f$ to 0s.

In the initializing stage, q uniform sampling sequences $y_{ci}\left[k\right],1\leq \;i\leq q,\;1\leq k\leq N_{0}$ can be obtained by classifying the periodic non-uniformly sampled data y[n], 1≤n≤N. Then the FFT of q uniform sampling sequences $y_{ci}\left[n\right]$ are calculated and expressed as $Y_{ci}\left[k\right],\;1\leq \;i\leq q,\;1\leq k\leq N_{0}$.

The first step achieves the function of compensating the fractional delay of q uniform sampling sequences $y_{ci}\left[n\right]$ in the frequency domain. Compared with the traditional method which uses the fractional delay filter to compensate the fractional delay, this method has lower computational complexity. Detailed analysis of the reduction of computational complexity is beyond the scope of this paper.

In the second step, let $N_{s}$ denotes the interval of choosing partial frequency bins from all bins uniformly. The frequency-smoothed cross-periodogram of $Y_{i}\left[m\right]$ and $Y_{j}\left[m\right]$ at partial frequency bins $m=lN_{s},1\leq \;l\leq N_{0}/N_{s}$ is calculated. We assume that $N_{0}$ is an integer multiple of $N_{s}$. Define $W_{\min}$ as the minimum modulation bandwidth of primary user, and △f as the bandwidth of a frequency bin. The width of the spectral window function w[n] and $N_{s}$ are both chosen to be $W_{\min}/△f$ to obtain a smoothed version of the original power spectrum. Please note that the width of the spectral window function is very important to obtain the outline information of the original power spectrum.

The third step uses the least square method to estimate the power spectrum at partial frequency bins ${\hat{\tilde{r}}}_{x}\left[m\right]$, which only needs repeatedly solving Equation (25) $N_{0}/N_{s}$ times. Compared with the traditional method which needs repeatedly solving Equation (25) $N_{0}$ times, the amount of calculation has been reduced by $N_{s}$ times.

Finally, the estimated power spectrum at partial frequency bins ${\hat{\tilde{r}}}_{x}\left[m\right]$ are compared with the predefined threshold γ. A sequence of binary results can be obtained after the comparison. The minimum modulation width of primary user $W_{\min}$ and the width of the frequency bin △f can be exploited to further refine the binary sequence. Consecutive 1s with length less than $W_{\min}/△f$ are flipped to 0s, then channels with 1s are considered occupied by primary users.

Some applications are sensitive to the computational complexity of the spectrum-sensing module, such as sensor nodes and battery-powered devices. Compared with the traditional method which estimates the power spectrum at all frequency bins, the proposed efficient spectrum-sensing algorithm only estimates power spectrum at partial frequency bins, by exploiting the frequency-smoothing characteristics of the power spectrum estimator. It is reasonable to use the power spectrum information at partial frequency bins to determine whether the primary user exists because the estimate result in the third step of Algorithm 1 is the weighted average result of the original power spectrum in a spectral window function.

## 5. Sampling Pattern Design

The matrix Φ is assumed to have full column rank when the least square method is used to estimate the power spectrum in Equation (25). In fact, whether it has full column rank is determined by the sampling pattern of MCS. Let us explore the structure of matrix $\Phi =\left(A^{*}⊗A\right)B$. Let $a_{i}^{*}$ and $a_{i},1\leq i\leq q$, denote the *i*-th row of matrix $A^{*}$ and A, respectively. $A^{*}$ and A can be further written as $A^{*}={\left(a_{1}^{H},a_{2}^{H},\ldots ,a_{q}^{H}\right)}^{T}$ and $A={\left(a_{1}^{T},a_{2}^{T},\ldots ,a_{q}^{T}\right)}^{T}$, respectively. The structure of $q^{2}\times L^{2}$ matrix $A^{*}⊗A$ is firstly explored and it can be written as:

(28) $$\begin{array}{lll} A^{*}⊗A & = & ({\left(a_{1}^{*}⊗a_{1}\right)}^{T},{\left(a_{1}^{*}⊗a_{2}\right)}^{T},\ldots ,{\left(a_{1}^{*}⊗a_{q}\right)}^{T},\\ & & \;{\left(a_{2}^{*}⊗a_{1}\right)}^{T},{\left(a_{2}^{*}⊗a_{2}\right)}^{T},\ldots ,{\left(a_{2}^{*}⊗a_{q}\right)}^{T},\\ & & \;\vdots \\ & & \;{\left(a_{q}^{*}⊗a_{1}\right)}^{T},{\left(a_{q}^{*}⊗a_{2}\right)}^{T},\ldots ,{\left(a_{q}^{*}⊗a_{q}\right)}^{T}{)}^{T}, \end{array}$$

where $a_{i}^{*}⊗a_{k},\;1\leq i,k\leq q$ is the (i−1)q+k-th row vector of matrix $A^{*}⊗A$. The $L^{2}\times L$ selection matrix B can be written as:

(29) $$B={\left(\begin{array}{lll} \begin{array}{ll} 1 & 0\\ 0 & 0 \end{array} & \cdots & \begin{array}{ll} 0 & 0\\ 0 & 0 \end{array}\\ \vdots & \ddots & \vdots \\ \begin{array}{ll} 0 & 0\\ 0 & 0 \end{array} & \cdots & \begin{array}{ll} 0 & 0\\ 0 & 0 \end{array} \end{array}\;\begin{array}{lll} \begin{array}{ll} 0 & 0\\ 0 & 1 \end{array} & \cdots & \begin{array}{ll} 0 & 0\\ 0 & 0 \end{array}\\ \vdots & \ddots & \vdots \\ \begin{array}{ll} 0 & 0\\ 0 & 0 \end{array} & \cdots & \begin{array}{ll} 0 & 0\\ 0 & 0 \end{array} \end{array}\;\ldots \;\begin{array}{lll} \begin{array}{ll} 0 & 0\\ 0 & 0 \end{array} & \cdots & \begin{array}{ll} 0 & 0\\ 0 & 0 \end{array}\\ \vdots & \ddots & \vdots \\ \begin{array}{ll} 0 & 0\\ 0 & 0 \end{array} & \cdots & \begin{array}{ll} 0 & 0\\ 0 & 1 \end{array} \end{array}\right)}^{T}.$$

Based on the structure of matrix B, it is not difficult to find that multiplying a $1\times L^{2}$ vector $a_{i}^{*}⊗a_{k}$ with a $L^{2}\times L$ selection matrix B is equivalent to $a_{i}^{*}.*a_{k}$, where .∗ denotes the matrix dot product. Therefore, matrix $\Phi =\left(A^{*}⊗A\right)B$ can be rewritten as:

(30) $$\begin{array}{lll} \left(A^{*}⊗A\right)B & = & ({\left(a_{1}^{*}.*a_{1}\right)}^{T},{\left(a_{1}^{*}.*a_{2}\right)}^{T},\ldots ,{\left(a_{1}^{*}.*a_{q}\right)}^{T},\\ & & \;{\left(a_{2}^{*}.*a_{1}\right)}^{T},{\left(a_{2}^{*}.*a_{2}\right)}^{T},\ldots ,{\left(a_{2}^{*}.*a_{q}\right)}^{T},\\ & & \;\vdots \\ & & \;{\left(a_{q}^{*}.*a_{1}\right)}^{T},{\left(a_{q}^{*}.*a_{2}\right)}^{T},\ldots ,{\left(a_{q}^{*}.*a_{q}\right)}^{T}{)}^{T}. \end{array}$$

Since the row vector $a_{i}$ of matrix A can be written as $a_{i}=\left[\exp\left(-j2\pi c_{i}\frac{1}{L}\right),\exp\left(-j2\pi c_{i}\frac{2}{L}\right),\ldots ,\exp\left(-j2\pi c_{i}\frac{L}{L}\right)\right]$, the (i−1)q+k-th row vector of matrix $\Phi =\left(A^{*}⊗A\right)B$ can be further written as

(31) $$a_{i}^{*}.*a_{k}=\left[\exp\left(-j2\pi \left(c_{k}-c_{i}\right)\frac{1}{L}\right),\exp\left(-j2\pi \left(c_{k}-c_{i}\right)\frac{2}{L}\right),\ldots ,\exp\left(-j2\pi \left(c_{k}-c_{i}\right)\frac{L}{L}\right)\right].$$

Please note that $a_{i}^{*}.*a_{k}$, the (i−1)q+k-th row of matrix Φ, has some special structure. It can be viewed as the $c_{k}-c_{i}+1$-th row of the L×L Discrete Fourier Transform (DFT) matrix when $c_{k}-c_{i}$ is greater than or equal to 0.

Matrix $\Phi =\left(A^{*}⊗A\right)B$ can be written as the product of two matrices using Equations (30) and (31),

(32) Φ=E×F,

where F is a L×L DFT matrix and E is a $q^{2}\times L$ matrix. The row vector of matrix E contains only one non-zero element 1 and the other elements are zero. The location of element 1 is $c_{k}-c_{i}+1$ when $c_{k}-c_{i}$ is greater than or equal to 0. The location of element 1 is $c_{k}-c_{i}+L$ when $c_{k}-c_{i}$ is less than 0. The row vector of matrix E acts similar to a selection vector, and it selects which row vector of the DFT matrix F as the current row vector of Φ. Obviously, the matrix Φ has full column rank as long as the matrix E has full column rank since the DFT matrix F is a full rank square matrix.

**Figure 4:**
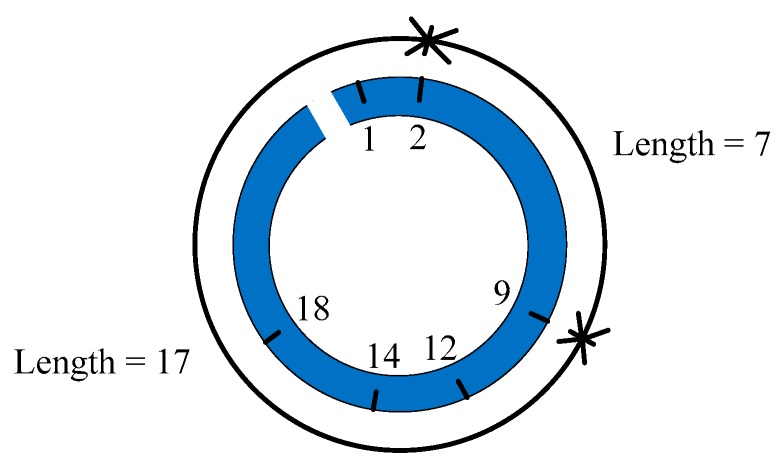
A length-24 circular sparse ruler.

Next, we briefly introduce the concept of the circular sparse ruler. A *L*-length circular sparse ruler can be viewed as a ruler with only q marks which is less than *L*, but it is also able to measure all the length between 1 and *L*. Define the q marks as $C=\left\{c_{1},c_{2},\ldots ,c_{q}\right\},\;C\subset \left\{1,2,\ldots ,L\right\}$, and their difference set $\Omega \left(C\right)=\left\{\left(c_{k}-c_{i}\right)\mathrm{mod}\;L|\forall c_{k},c_{i}\in C\right\}$, where (·mod·) is the modulus operator. A set C is a circular sparse ruler if its difference set Ω(C)={1,2,…,L}. An example of the circular sparse ruler is given in Figure 4, where the marks are C={1,2,9,12,14,18} and the measurement range is L=24. Although there is no mark 7 and 17 on the sparse ruler, length 7 and 17 can be measured using mark 2 and 9.

The definition of difference set of circular sparse ruler $\Omega \left(C\right)=\left\{\left(c_{k}-c_{i}\right)\mathrm{mod}\;L|\forall c_{k},c_{i}\in C\right\}$ can be rewritten as $\Omega \left(C\right)=\Omega_{1}\left(C\right)\cup \Omega_{2}\left(C\right)$, where $\Omega_{1}\left(C\right)=\left\{c_{k}-c_{i}+1\;|\forall c_{k},c_{i}\in C\;\mathrm{and}\;c_{k}-c_{i}\geq 0\right\}$ and $\Omega_{2}\left(C\right)=\left\{c_{k}-c_{i}+L\;|\forall c_{k},c_{i}\in C\;\mathrm{and}\;c_{k}-c_{i}<0\right\}$. That is, if the difference set of the sampling pattern of MCS includes the set {1,2,…,L}, then each column vector of matrix E contains at least one non-zero element 1. Combined with the fact that the row vector of E has only one non-zero element 1, the matrix E will have full column rank. Therefore, the sampling pattern design of the MCS can be viewed as a circular sparse ruler problem. Please note that circular sparse ruler uses the difference between two marks to measure any length between 1 and L. If we want to measure length 1 to length L, then at least one pair of marks, or cosets, should have length 1 difference, or 1×T time difference. Further, to obtain the strongest compression rate, we would like to minimize the cardinality of C, which can be viewed as a minimal circular sparse ruler problem. Then finally, let $c_{i+2}$ and $c_{i}$ denote the *i*+2-th and *i*-th coset, respectively. $c_{i+2}$ should be greater than $c_{i}+d$ because the S/H in Figure 2 can only temporarily store one sample and the minimal sampling interval of ADC is $T_{\mathrm{ADC}}=d\times T$. Therefore, the design of sampling pattern turns into the following optimization problem:

(33) $$\underset{C}{\min}\left|C\right|\;s.t.\;\{\begin{array}{l} \Omega \left(C\right)=\left\{1,2,\ldots ,L\right\}\\ c_{i+2}>c_{i}+d,\;1\leq i\leq q-2 \end{array},$$

where the sampling pattern C⊂{1,2,…,L}.

## 6. Simulation Results

This section evaluates the spectrum-sensing performance of the proposed single-channel sub-Nyquist sampling by performing several numerical simulations. In practice, an antenna is used to receive the Radio Frequency (RF) signal, and a band-pass filter is utilized to filter the out-of-band noise and interference. Subsequently, the RF signal is demodulated to baseband signal using a mixer. Finally, the Local Oscillator (LO) signal mixed in the demodulated signal is filtered out using a low-pass filter. Since the receiver front-end circuit is not the focus of this paper, we assume that the proposed single-channel sub-Nyquist sampling directly deals with the demodulated signal output from the low-pass filter in Figure 2. Quadrature Phase-Shift Keying (QPSK) signal is used as the test signal, and it is generated by the following model:

(34) $$\begin{array}{lll} x\left(t\right) & = & s\left(t\right)+n\left(t\right)\\ & = & E\sum_{k=1}^{M}\left\{\sum_{i=1}^{N_{b}}I_{k}\left[i\right]g_{k}\left(t-iT_{k}\right)\cos\left(2\pi f_{k}t\right)+\sum_{j=1}^{N_{b}}Q_{k}\left[j\right]g_{k}\left(t-jT_{k}\right)\sin\left(2\pi f_{k}t\right)\right\}+n\left(t\right), \end{array}$$

where M is the number of primary users, $N_{b}$ is the number of random bits, $I_{k}\left[i\right]$ and $Q_{k}\left[j\right]$ are random bit streams, $g_{k}\left(t\right)$ is the pulse-shaping function, $T_{k}$ is the symbol duration, $f_{k}$ is the carrier frequency of the *k*-th primary user, and n(t) is the additive white Gaussian noise. Simulation parameters are set as follows:

1. The bandwidth of primary user is set as $\frac{1}{T_{k}}=8\;\mathrm{MHz}$. The carrier frequency of primary user is randomly chosen from the center frequency of 40 channels. The pulse-shaping function is the root-raised cosine with roll-over factor 0.1. $I_{k}\left[i\right]$ and $Q_{k}\left[j\right]$ are generated from pseudo-random binary sequence generator.
2. Additive white Gaussian noise $n\left(t\right)\sim N\left(0,\sigma^{2}\right)$, where $\sigma^{2}$ is fixed to 1 and E in Equation (34) is scaled to a certain SNR level.
3. The minimum modulation bandwidth of primary user is set as $W_{\min}=500\;\mathrm{KHz}$.
4. To simulate the mismatch behavior of parallel MCS, the clock timing skew is set as 2% of the Nyquist sampling interval T. The offset and gain mismatch are set as 2% and 2% of maximum amplitude of signal, respectively.
5. The parameters of log-normal shadowing in Equation (2) are set as follows: reference distance $d_{0}=1\;\mathrm{km}$, the path loss at the reference point $PL_{0}=0\;\mathrm{dB}$, the path loss exponent γ=2.4, the Gaussian random variable $X_{\rho }\sim N\left(0,5\right)\;\mathrm{dB}$, the distance between the primary user and the cognitive radio device d~N(5,3) km. In addition, primary users are passed through Rayleigh channels with $2\lambda^{2}=2\;\mathrm{MHz}$.
6. In the second and third numerical simulation, 200 trials are performed, and averaged results are shown.

**Figure 5:**
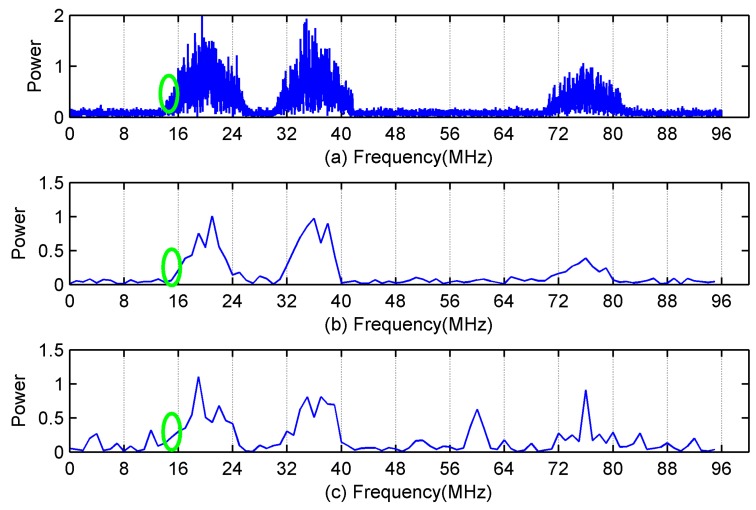
(**a**) Original power spectrum. (**b**) Estimated power spectrum at partial frequency bins using the proposed serial MCS. (**c**) Estimated power spectrum at partial frequency bins using the traditional parallel MCS. The green circle represents the Doppler shift caused by the multipath effect.

In the first simulation, we test the feasibility of spectrum sensing using power spectrum at partial frequency bins. To show the estimated frequency-smoothed power spectrum clearly, we only observed 12 of these 40 channels. In the following two simulations, 32 of these 40 channels are observed. The parameters of MCS are set as L=24, q=6 and the sampling pattern is C={1,2,9,12,14,18}. The number of active primary users is set as M=3. The SNR of the test signal is set as 10 dB, and the acquisition time is set as 64 μS. Figure 5a shows the original power spectrum of the test signal. Figure 5b,c show the estimated frequency-smoothed power spectrum at partial frequency bins using the proposed serial MCS and the traditional parallel MCS, respectively.

Figure 5b reflects the profile information of the original power spectrum because frequency-smoothed cross-periodogram is used in Equation (24). Using the frequency-smoothed cross-periodogram is equivalent to taking a weighted average of the power spectrum in the frequency-smoothing window. The weight value in the middle of the window is the largest and decreases toward each side. The profile information of the original power spectrum is sufficient for the spectrum-sensing module to detect primary users. It is unnecessary to estimate power spectrum at all frequency bins because the power spectrum at two frequency bins within the frequency-smoothing window are highly correlated. The proposed efficient spectrum-sensing algorithm exploits the frequency-smoothing characteristic to reduce the computational complexity.

On the other hand, as shown in Figure 5c, mismatches among sub-ADCs in traditional parallel MCS cause large errors in the estimated power spectrum. Although these errors do not exist after calibration, the calibration will add a lot of additional burden to the system. Therefore, the proposed serial MCS combined with the efficient spectrum-sensing algorithm is a better choice.

In practice, the primary user signal arrives at the antenna of the cognitive radio device through multiple paths. Due to the different arrival time, the superposition of these signals will result in the distortion of the original signal. Rayleigh distribution is a model that can be used to describe this form of fading when multipath propagation exists. Rayleigh fading will result in Doppler shift, which equivalents to the expansion in the frequency domain. As shown in the green circle of Figure 5, although the baseband width of the primary user of the third channel is 8 MHz, the Doppler shift caused by the multipath effect causes the energy of the third channel leak into the second channel. As a result, cognitive radio devices may mistakenly regard the primary user of the second channel also exists. In addition, Log-normal Shadowing significantly attenuates the amplitude of signal. When the amplitude of primary user signal is close to that of noise, the proposed method is difficult to detect the presence of the primary user. Cooperative spectrum sensing based on decision fusion can efficiently solve the shadow fading problem.

In the second simulation, we evaluate spectrum-sensing performance in terms of different compression ratio. 32 channels are observed in this simulation. Its bandwidth and Nyquist sampling rate are 256 MHz and 512 MSPS (Mega-Samples Per Second), respectively. We consider several SNRs and they are 5 dB, 0 dB, −5 dB and −10 dB. The periodicity of the sampling instants is set as L=64 in MCS. Several compression ratio are considered, and they are 0.25, 0.375 and 0.5. The sampling pattern for compression ratio 0.25 is C={1,5,9,10,14,18,25,26,33,37,38,43,49,51,54,61}, and the sampling rate requirement for ADC is 128 MSPS. The sampling pattern for compression ratio 0.375 is C={1,3,5,7,9,10,12,14,16,18,21,25,26,29,33,37,38,41,43,45,49,51,54,61}, and the sampling rate requirement for ADC is 192 MSPS. The sampling pattern for compression ratio 0.5 is C={1,3,5,7,9,10,12,14,16,18,19,21,25,26,27,29,33,35,37,38,39,41,42,43,45,47,49,51,53,54,61,62}, and the sampling rate requirement for ADC is 256 MSPS. The number of active primary users is set as M=8. The acquisition time is set as 64 μS.

**Figure 6:**
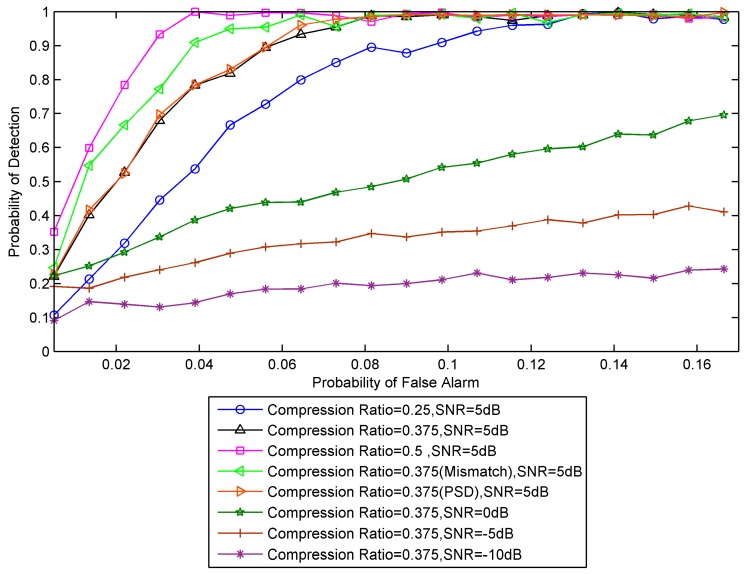
Influence of compression ratio on the ROC.

Figure 6 shows the Receiver Operating Characteristic (ROC) curve of the detector for different compression ratios. It is noticed that spectrum-sensing performance under different compression ratios is different. The more compression, the worse the detection performance. The curve corresponding to ‘Compression Ratio = 0.375 (Mismatch)’ in Figure 6 is spectrum-sensing performance based on the parallel MCS with mismatches. Please note that the detection performance of the parallel MCS is better than that of the proposed serial MCS. The reason is that the mismatch error component is mistakenly regarded as primary users. The curve corresponding to ‘Compression Ratio = 0.375 (PSD)’ in Figure 6 is spectrum-sensing performance using the power spectrum at all frequency bins. The detection performance is slightly different from the detection performance using the power spectrum at partial frequency bins, while the later one reduces a large amount of computation. Table 1 shows the reduced computational complexity. The proposed efficient spectrum-sensing algorithm uses the frequency-smoothed cyclic cross-periodogram to estimate the smoothed power spectrum. Since the power spectrum of the smoothed power spectrum at two adjacent frequency bins are highly correlated, it is not necessary to estimate the power spectrum at all frequency bins, which reduces the computational complexity of spectrum sensing.

In the third simulation, we evaluate spectrum-sensing performance in terms of different acquisition time. The proposed efficient spectrum-sensing algorithm requires the signal be stationary during the acquisition process. To consider the fast time-varying signal, the detection performance under short acquisition time condition is tested. The compression ratio is fixed to 0.375. Several acquisition time are considered, and they are 16 μS, 64 μS, and 128 μS. Other simulation parameters are the same with the second simulation.

**Figure 7:**
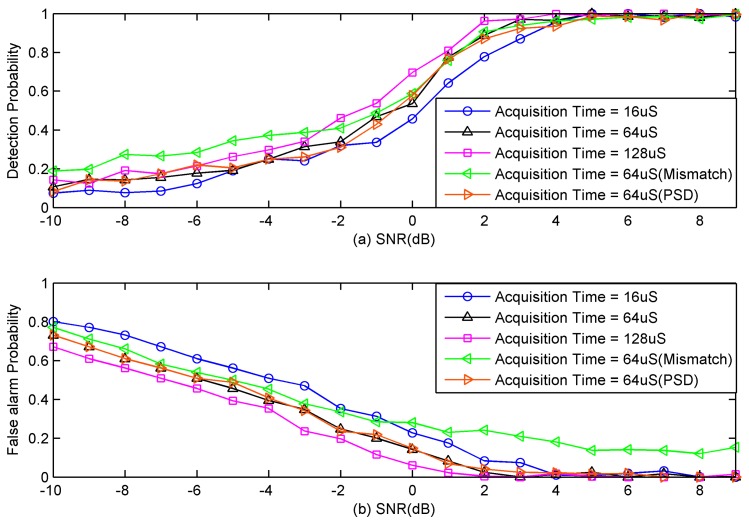
Influence of acquisition time on spectrum-sensing performance. (**a**) Detection probability. (**b**) False alarm probability.

As shown in Figure 7, spectrum-sensing performance gradually improves as the SNR increases. When the SNR is greater than 5 dB, the proposed efficient spectrum-sensing algorithm exhibits a perfect detection performance. The shorter the acquisition time, the worse the spectrum-sensing performance. Short acquisition time means that the number of samples used to calculate frequency-smoothed cross-periodogram in Equation (24) is small. Thus, the estimate accuracy of the frequency-smoothed cross-periodogram is affected. Ultimately, spectrum-sensing performance is influenced. In the case that the acquisition time is 16 μS, it is guaranteed that all primary users and spectrum holes can be detected with high probability when the SNR is higher than 5 dB.

The curve corresponding to ‘Acquisition Time = 64 μS (PSD)’ is spectrum-sensing performance using power spectrum at all frequency bins. It is shown that spectrum-sensing performance based on power spectrum at all frequency bins has little improvement. Therefore, spectrum sensing using power spectrum at partial frequency bins is preferable since it reduces a large amount of computation. Table 2 compares the required computational complexity between the conventional method and the proposed method. The reason for the reduction of computational complexity is the same as that of the second simulation.

Compared with the detection performance, the false alarm probability is also very important. False alarm probability determines how many spectrum holes are available to secondary users. The curve corresponding to ‘Acquisition Time = 64 μS (Mismatch)’ is spectrum-sensing performance using the traditional parallel MCS with mismatches. Mismatches have little influence on the detection performance as shown in Figure 7a, while they have severe influence on the false alarm probability as shown in Figure 7b. The false alarm probability of the proposed serial MCS gradually decreases to 0 as the SNR increases. However, the false alarm probability of the traditional parallel MCS does not tend to zero even if the SNR is high. As shown in Figure 7b, all spectral holes can be detected with high probability when the SNR is higher than 8dB for serial MCS.

## 7. Discussion

The periodic non-uniformly sampled data obtained by the proposed serial MCS can be classified into several uniform sampling sequences. Waveform reconstruction can be achieved using MCS algorithm and compressive sensing [8]. The reconstructed waveform can be used for decoding, so the spectrum-sensing module can also be used as a receiver. The alternative function of the spectrum-sensing module saves hardware cost in terms of price and size. It is worth noting that the sampling pattern design for waveform reconstruction is different from the sampling pattern design for power spectrum estimation proposed in this paper, so the sampling pattern design should be given additional consideration.

## 8. Conclusions

This paper proposes a single-channel sub-Nyquist sampling structure, serial MCS, for wideband spectrum sensing. Although the requirement for the sampling rate of ADC is higher compared with the traditional parallel MCS, the proposed serial MCS cannot only avoid the mismatch among sub-ADCs in traditional parallel MCS, but also reduce the hardware implementation size. An efficient spectrum-sensing algorithm based on power spectrum at partial frequency bins is also developed, which could save a large amount of computational cost. A sampling pattern design method for the proposed serial MCS is given as well. Simulations show that the mismatch in traditional MCS has a serious impact on spectrum-sensing performance, and the proposed MCS combined with the efficient spectrum-sensing algorithm exhibits outstanding spectrum-sensing performance at much lower computational cost.

## Figures and Tables

**Table 1:** Computational Complexity Required.

Compression Ratio	Conventional	Proposed	Saved
0.25	512 ^1^	32	93.75%
0.33	512	32	93.75%
0.5	512	32	93.75%

^1^ The unit is the number of solving the Least Square Equation.

**Table 2:** Computational Complexity Required.

Acquisition Time	Conventional	Proposed	Saved
16 μS	128 ^1^	8	93.75%
64 μS	512	32	93.75%
128 μS	1024	64	93.75%

^1^ The unit is the number of solving the Least Square Equation.
